# Leucine‐Restricted Diet Attenuates Small Intestinal Tumorigenesis in Apc^Min^ ^/+^ Mice

**DOI:** 10.1002/fsn3.72100

**Published:** 2026-07-09

**Authors:** Yunlong Sui, Namiko Hoshi, Norihiro Okamoto, Yuna Ku, Makoto Ooi, Daisuke Watanabe, Haruka Miyazaki, Misaki Agawa, Hirotaka Nakamura, Jun Inoue, Hui Yang, Yuzo Kodama

**Affiliations:** ^1^ Department of Gastroenterology The Second Affiliated Hospital of Guangzhou Medical University Guangzhou Guangdong China; ^2^ Division of Gastroenterology, Department of Internal Medicine Kobe University Graduate School of Medicine Hyogo Japan

**Keywords:** amino acids, intestinal tumors, mismatch repair, mTORC1

## Abstract

Red and processed meats have been implicated in intestinal tumorigenesis; however, the roles of their main nutritional components, amino acids, in the modulation of adenoma development remain unclear. This study aimed to investigate the roles of various amino acids in intestinal tumorigenesis. Apc^Min/+^ mice, as a model of spontaneous intestinal adenoma, were fed diets restricted to essential amino acids (leucine, lysine, valine), or the non‐essential amino acid arginine to observe the modulation of tumorigenesis. The mice were fed diets with a reduction in each amino acid starting at 5 weeks of age and analyzed at 15 weeks of age. Among the diets tested, only the 90% leucine‐restricted (Leu‐R) diet dramatically reduced the size and number of small intestinal tumors. Mechanistically, both cell proliferation and mTORC1 pathway activation were reduced in tumors from the 90% Leu‐R group. Further, Gene Set Enrichment Analysis (GSEA) showed that the 90% Leu‐R diet affected pathways related to DNA repair and genome stability in small intestinal tumors. Notably, mismatch repair (MMR) genes, *Mlh1*, *Msh2*, and *Pms2*, were upregulated in small intestinal tumors from the 90% Leu‐R group. Organoids derived from small intestinal tumors from the 90% Leu‐R group exhibited fewer and smaller spherical structures. Leucine plays a key role in small intestinal tumorigenesis in Apc^Min/+^ mice. The 90% Leu‐R diet suppressed activation of the mTORC1 pathway and maintained genomic stability presumably via the MMR system. These findings suggest the potential for tumor prevention strategies targeting specific essential amino acids.

## Graphical abstract

Essential amino acids have been implicated in intestinal physiology and disease, but their individual roles in intestinal tumorigenesis remain unclear. Among the amino acids examined, dietary leucine restriction uniquely suppressed small intestinal tumorigenesis in Apc^Min/+^ mice. This effect was accompanied by reduced mTORC1 signaling and increased expression of mismatch repair genes, suggesting a potential role in maintaining genomic stability and limiting tumor cell proliferation.

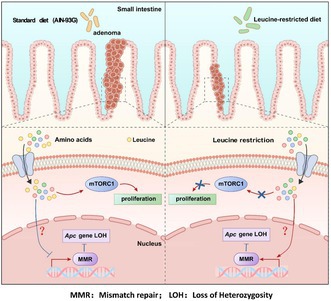

## 1 Introduction

Epidemiological studies show that the risk of tumor development in both the small and large intestines is increasing (Eng et al. 2024; Qubaiah et al. 2010), posing a significant challenge to healthcare systems. Intestinal adenomas, characterized by abnormal proliferative lesions originating from intestinal epithelial cells, are primary precancerous lesions (Lu et al. 2024). Therefore, proactive prevention of intestinal adenomas is crucial to control and reduce the incidence of intestinal tumors, which may alleviate the substantial burden on public health systems.

Dietary interventions have emerged as a research focus in the pursuit of preventive strategies for intestinal tumors (Hull 2021). A positive correlation has been indicated between the excessive consumption of red or processed meat and the development of intestinal tumors (Abu‐Ghazaleh et al. 2021; GBD Colorectal Cancer Collaborators 2022). Essential amino acids—those that cannot be synthesized endogenously and must be obtained through the diet—are present at relatively high levels in meat and have been implicated to play some roles in intestinal physiology and disease. For instance, leucine levels are associated with intestinal stem cell activity, inflammatory bowel conditions, and colorectal cancer (Lu et al. 2021; Ravindran et al. 2016; Wang et al. 2022); increased dietary intake of lysine may alleviate intestinal inflammation and irritable bowel syndrome (IBS)‐like symptoms (Smriga and Torii 2003; Tang et al. 2025). However, the specific roles of individual amino acids in intestinal adenoma development remain unclear.

We, therefore, used Apc^Min/+^ mice, a well‐established mouse model of spontaneous intestinal adenoma, and fed them amino acid‐restricted diets to assess the effects of individual amino acid deficiencies. Our study demonstrates the importance of amino acids, especially leucine, in intestinal tumorigenesis and provides a theoretical foundation for intestinal tumors prevention strategies using dietary amino acid modulation.

## 2 Materials and Methods

### 2.1 Animals and Diets

All animal experiments were approved by the Institutional Animal Care and Use Committee of Kobe University (approval number: P190306). Mice were bred in a specific pathogen‐free environment at the Animal Facility of Kobe University Graduate School of Medicine. C57BL/6J (B6) mice were purchased from SLC (Japan), and Apc^Min/+^ mice on a C57BL/6J background (strain #002020) were purchased from Jackson Laboratory (Bar Harbor, ME, USA), and both male and female mice were used in this study.

AIN‐93G (containing 1.59% leucine [w/w]), a standard rodent diet formulated by the American Institute of Nutrition (AIN) in 1993 for nutritional research, was used as the control diet. Special diets with varying amino acid content were obtained from Oriental Yeast Co. Ltd. (Japan). The leucine‐restricted (Leu‐R) diets contained 0.159%, 0.477%, and 0.795% leucine (w/w), corresponding to reductions of 90%, 70%, and 50% in leucine content, respectively. Lysine‐restricted (Lys‐R), arginine‐restricted (Arg‐R), and valine‐restricted (Val‐R) diets were also formulated. The detailed diet composition is presented in Table S1.

### 2.2 Animal Experimental Design

For tumor analysis: Age‐ and sex‐matched Apc^Min/+^ littermates were randomly assigned to the control and experimental groups. The control group was fed AIN‐93G, whereas experimental groups were fed the specific amino acid‐restricted diets described above. Because the AIN‐93G diet served as the common control diet for comparison with different amino acid‐restricted diets, AIN control mice from comparable experimental batches conducted under the same experimental conditions were pooled for the final statistical analysis.

In the early intervention experiment, dietary treatment was initiated at 5 weeks of age and continued until 15 weeks of age. In the late‐intervention experiment, dietary treatment was initiated at 12 weeks of age and continued until 15 weeks of age. At 15 weeks of age, the small intestine and colon were excised and opened longitudinally. The same researcher, blinded to the diet condition, assessed the tumor size and number throughout the study.

For body weight and food intake analysis: B6 mice were housed and administered the diets in the same manner as described for the Apc^Min/+^ experiment. Body weight was recorded weekly from 5 to 15 weeks of age, and food intake was also measured weekly.

### 2.3 Histology

Normal and tumor tissues from the small intestine were harvested, fixed in formalin (#133–10,311; Fujifilm, Wako, Osaka, Japan), and embedded in paraffin. Hematoxylin and eosin (HE) staining was used to analyze the number of intestinal Paneth cells according to the following method: three intact crypts were randomly selected from each sample, and Paneth cells were manually counted under a microscope (×400 magnification) by a researcher blinded to the mouse genotypes. The average number of Paneth cells per crypt was calculated and used for data analysis. For immunohistochemistry, Ki‐67 (1:100; #12202; Cell Signaling Technology, Danvers, MA), p‐S6 (1:200; #4858S; Cell Signaling Technology), and Olfm4 (1:200; #39141; Cell Signaling Technology) were used as primary antibodies, and the ABC HRP Kit Peroxidase (#pk6101; Vector Laboratories, Newark, CA) was used as the secondary antibody. ImageJ software (National Institutes of Health, Bethesda, MD) was used to analyze Ki‐67 positive cells by researchers who were blinded to the mouse genotypes. 3,3‐Diaminobenzidine (DAB) was used as the chromogenic substrate for p‐S6 immunohistochemical staining. Images of p‐S6‐immunostained sections were acquired using Olympus cellSens Standard (version 4.2). Digital images were exported in TIFF format and imported into Fiji (ImageJ, National Institutes of Health, Bethesda, MD, USA). For quantification of staining intensity, color deconvolution was performed using the Color Deconvolution plugin with the H DAB vector setting. The DAB channel image was subsequently inverted prior to intensity measurement so that higher pixel values corresponded to stronger DAB staining. Regions of interest (ROIs) were manually defined to include the tumor area while excluding background regions. Mean intensity values within each ROI were measured and used for subsequent analysis. Quantitative image analysis was performed in a blinded manner.

### 2.4 RNA Extraction and Real‐Time Polymerase Chain Reaction (PCR)

Small intestinal normal tissue and tumors were harvested and preserved in RNAlater (#AM7021; Thermo Fisher Scientific, Waltham, MA) until use. Total RNA was extracted using TRIzol reagent (#15596018; Thermo Fisher Scientific) and reverse‐transcribed into cDNA using a High‐Capacity cDNA Reverse Transcription Kit (#4374967; Applied Biosystems, Foster City, CA) following the manufacturer's instructions. Real‐time PCR was performed using SYBR Green (#4367659; Applied Biosystems) on a QuantStudio3 PCR system (Applied Biosystems), and the cycling conditions were as follows: an initial denaturation at 95°C for 10 min, followed by 40 cycles of denaturation at 95°C for 15 s and annealing at 60°C for 60 s. The relative expression levels of the target genes were measured using the relative standard curve method as previously described (Sui et al. 2025), and normalized to the expression of hypoxanthine‐guanine phosphoribosyltransferase (*Hprt*). The primer sequences are listed in Table 1.

**TABLE 1:** Primers used for real‐time PCR in this study.

Gene	Forward (5′‐3′)	Reverse (5′‐3′)
*Defa4*	CCAGGGGAAGATGACCAGGCTG	TGCAGCGACGATTTCTACAAAGGC
*Defa5*	AGGCTGATCCTATCCACAAAACAG	TGAAGAGCAGACCCTTCTTGGC
*Lyz1*	GCCAAGGTCTACAATCGTTGTGAGTTG	CAGTCAGCCAGCTTGACACCACG
*Wnt3*	TAAAGTGTAAATGCCACGGGTT	CGGAGGCACTGTCGTACTTG
*Lgr5*	ACCTGTGGCTAGATGACAATGC	TCCAAAGGCGTAGTCTGCTAT
*Mlh1*	GTTTTACTCCATTCGGAAGCAGT	TGTGAGCGGAAGGCTTTATAGAT
*Msh2*	GTGCAGCCTAAGGAGACGC	CTGGGTCTTGAACACCTCGC
*Msh6*	GGCTGGGTTAGCAAAAGGATG	TAAGCCTCATGCACCTCTGTC
*Pms2*	CGCAGGTTGAAACTTTTGGCT	CCGTGGCAGGTAGATATAGTGA
*Hprt*	GTTGGATACAGGCCAGACTTTGTTG	CCAGTTTCACTAATGACACAAACG

### 2.5 Western Blotting

Normal small intestinal tissue proteins were extracted with radioimmunoprecipitation buffer, and their concentration was determined using a bicinchoninic acid protein assay kit (#23227; Thermo Fisher Scientific). The proteins were separated by sodium dodecyl sulfate‐polyacrylamide gel electrophoresis (10% for β‐actin and Wnt3). The following primary antibodies were used: Wnt3 (1:1000, #ab172612; Abcam, Cambridge, UK), β‐actin (1:2000; #8457; Cell Signaling). After incubation with the primary antibodies, the membranes were incubated with an anti‐rabbit IgG (H + L) secondary antibody (1:5000; #31458; Thermo Fisher Scientific). The chemiluminescent signals were captured using an ImageQuant LAS 4000 Mini Imager (GE Healthcare, Chicago, IL). The results were obtained using ImageQuant TL software (GE Healthcare), and the band intensities were measured using ImageJ software (NIH).

### 2.6 RNA Sequencing

RNA from small intestinal tumors was extracted as described previously. RNA quality was assessed using a 5200 Fragment Analyzer System (Agilent Technologies, Santa Clara, CA), ensuring an RNA Quality Number > 8. Library construction and data processing were performed at the Bioengineering Laboratory (Sagamihara, Japan). The libraries were sequenced on a DNBSEQ‐G400 platform (MGI Tech, China) in paired‐end 150 bp (PE150) mode, generating 24–27 million read pairs per sample. Sequencing quality was evaluated using Phred quality scores (Q30), with Q30 > 85%. After quality filtering using Cutadapt (version 1.9.1) and Sickle (version 1.33), sequence alignment was conducted using Hisat2 (version 2.2.0) software, using the GCF_000001635.26 ( *Mus musculus* reference genome), sourced from the NCBI Genome Database (https://www.ncbi.nlm.nih.gov/datasets/genome/GCF_000001635.26).

### 2.7 Functional Enrichment Analysis

Normalized gene expression data in TPM format were analyzed by Gene Set Enrichment Analysis (GSEA) as previously described (Subramanian et al. 2005). Gene set files were downloaded from the Molecular Signatures Database (MSigDB), including the Hallmark collection (mh.all.v2024.1.Mm.symbols.gmt), and from the Kyoto Encyclopedia of Genes and Genomes (KEGG) database. Significant enrichment was defined as |normalized enrichment score (NES)| > 1 and false discovery rate (FDR) < 0.25.

### 2.8 Measurement of Amino Acid Levels in Plasma via Liquid Chromatography–Mass Spectrometry

Blood samples were collected from 11‐week‐old B6 mice that had been fed with 90% Leu‐R and AIN‐93G for 3 weeks. Plasma amino acid levels were measured as previously described (Sui et al. 2023). Briefly, 10 μL of plasma was mixed with 10 μL of internal standard (#293–73,701, WAKO), followed by incubation with 1 mL of methanol on ice. After centrifugation, the supernatant was analyzed on a Q‐Trap 6500 (Sciex) coupled to a Shimadzu LC‐30 ad high‐performance liquid chromatography system using an Intrada Amino Acid column (100 mm × 3.0 mm, 3.0 μm, Imtakt). Amino acids were separated using a gradient of acetonitrile/formic acid/100 mM ammonium formate and quantified by multiple reaction monitoring.

### 2.9 Organoid Culture and Analysis

Intestinal organoid culture and analysis were performed as previously described (Sui et al. 2023). Briefly, the small intestinal tumors were excised and incubated in PBS containing 2.5 mM EDTA on a rotator at 4°C for 30 min, then transferred to 45 mL PBS containing 10% FBS, vortexed, and filtered. IntestiCult organoid growth medium (#06005; STEMCELL Technologies, Vancouver, BC, Canada) and Matrigel (#356231; Corning, Corning, NY) were used for organoid culture. Organoids were counted blindly on day 5 at 40× magnification. Five spherical (Apc^Min/+^) organoids were randomly selected, and the calculated average diameter was used as the spherical organoid diameter.

### 2.10 Statistical Analysis

Data are presented as mean ± standard error of the mean (SEM) or median with interquartile range (IQR). Prism 9 (GraphPad Software Inc., La Jolla, CA) was used for the analysis. For comparisons between two groups, normally distributed data with equal variances were analyzed using an unpaired two‐tailed Student's *t*‐test, whereas non‐normally distributed data were analyzed using the Mann–Whitney U test. For comparisons involving more than two groups, one‐way analysis of variance (ANOVA) followed by Tukey's multiple‐comparisons test was used when the data were normally distributed, whereas the Kruskal–Wallis test followed by Dunn's multiple‐comparisons test was used when the data were not normally distributed. Tumor incidence, defined as the proportion of mice with tumor versus tumor‐free mice, was analyzed using Fisher's exact test. Statistical significance was set at *p* < 0.05.

## 3 Results

### 3.1 A 90% Leucine‐Restricted Diet Significantly Reduces Small Intestinal Tumor Size and Number in Apc^Min^ ^/+^ Mice

We reviewed the amino acid content in meat and found that essential amino acids leucine and lysine are present in relatively high concentrations (Górska‐Warsewicz et al. 2018), suggesting their critical roles in biological processes. Additionally, the mTORC1 pathway is one of the most important nutrient‐sensing signaling pathways in cells, playing a central role in regulating cell growth and is frequently aberrantly activated in various cancers (Saxton and Sabatini 2017). Studies have shown that some individual amino acids, such as leucine, valine, and the non‐essential amino acid arginine, can regulate cell proliferation through the mTORC1 pathway (Dyachok et al. 2016).

**FIGURE 1:**
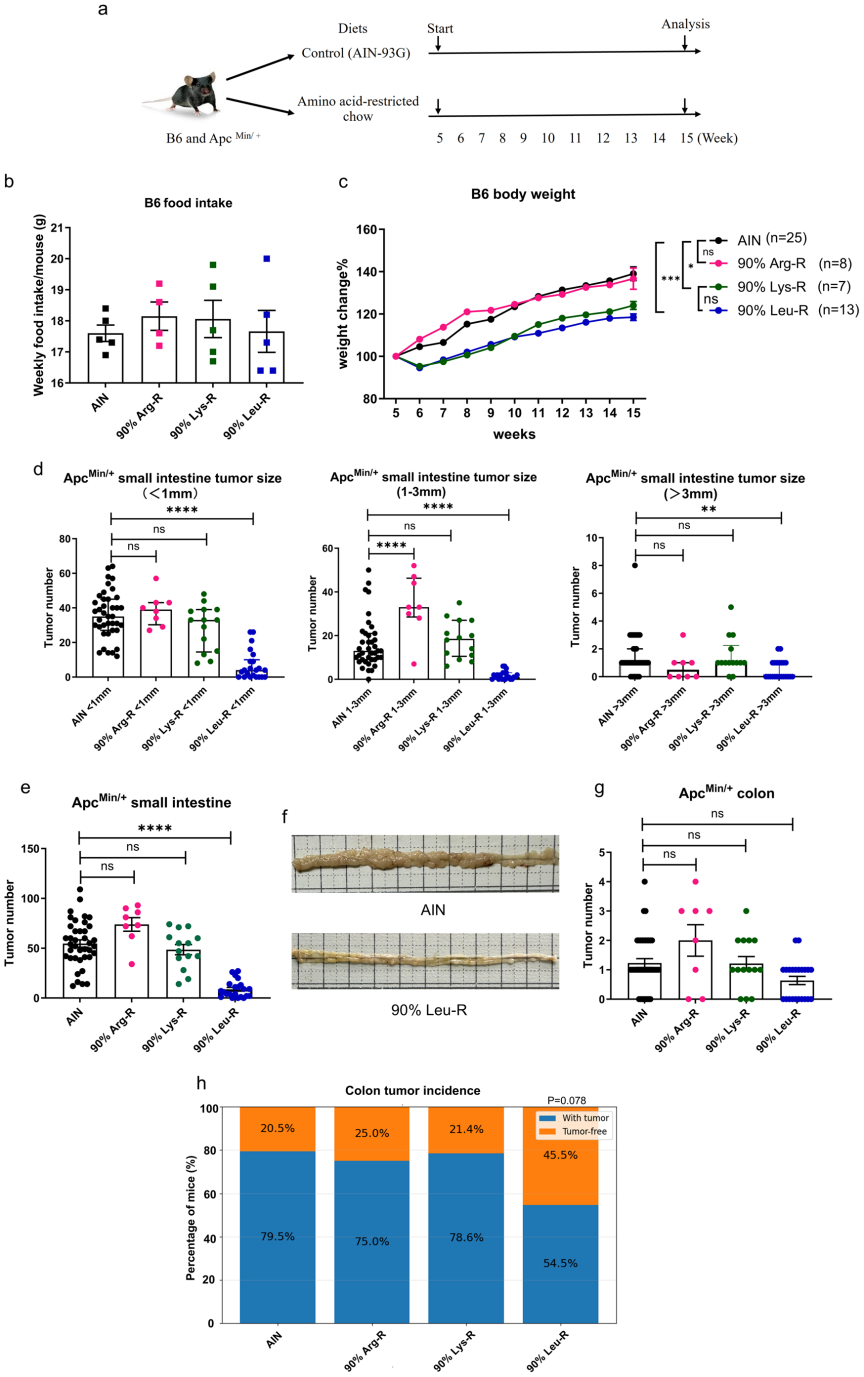
A 90% leucine‐restricted diet significantly reduces small intestinal tumor size and number in Apc^Min/+^ mice (a) Feeding schedule. (b) The weekly food intake. Mice were housed individually in single cages. (c) Body weight changes were shown, and statistical analysis was performed at week 15. (d) Tumor size and (e) number in the small intestine. (f) Representative images from the small intestine. (g) Tumor number in the colon. AIN (*n* = 39), 90% Arg‐R (*n* = 8), 90% Lys‐R (*n* = 14), 90% Leu‐R (*n* = 22). (h) Stacked bar graph showing the colon tumor incidence. Data in Figure 1d are presented as median with IQR, whereas other data (Figure 1b, 1c, 1e, 1g) are presented as mean ± SEM; statistical analysis was conducted using one‐way ANOVA followed by Tukey's multiple‐comparisons test (Figure b, c, e, g) or Kruskal–Wallis test followed by Dunn's multiple‐comparisons test (Figure d), Fisher's exact test was used for Figure h. Statistical significance levels are indicated as follows: *p < 0.05, **p < 0.01, ****p < 0.0001; ns (not significant), *p* > 0.05.

To explore their physiological impact, we first examined the effects of these amino acids in wild‐type B6 mice. Mice were fed diets with a reduction in each amino acid compared to the AIN‐93G standard diet, starting at 5 weeks of age and analyzed at 15 weeks of age (Figure 1a). The results showed that the 90% Leu‐R, Lys‐R, and Arg‐R diets did not affect food intake (Figure 1b: F (3,15) =0.27, *p* = 0.85). However, the 90% Leu‐R and Lys‐R diets significantly reduced body weight compared to the control group (Figure 1c: F (3,49) =8.3, *p* = 0.0001; AIN vs. 90% Lys‐R, *p* = 0.046; AIN vs. 90% Leu‐R, *p* = 0.0002). In contrast, the 90% Val‐R diet significantly reduced appetite (Figure S1a: *p* = 0.0019) and body weight (Figure S1b: *p* < 0.0001), which led us to terminate the valine experiment.

We next investigated the role of these amino acids in intestinal tumorigenesis using Apc^Min/+^ mice. Among all tested diets, only the 90% Leu‐R diet significantly reduced the small intestinal tumor size and number (Figure 1d–f. Figure 1d: (< 1 mm) AIN vs. 90% Leu‐R, *p* < 0.0001; (1‐3 mm) AIN vs. 90% Leu‐R, p < 0.0001; (> 3 mm) AIN vs. 90% Leu‐R, *p* = 0.0049. Figure 1e: F (3,79) =35.61, *p* < 0.0001; AIN vs. 90% Leu‐R, p < 0.0001). To rule out the possibility that lysine reduction was insufficient to influence tumor size and number, we further decreased lysine content (95% Lys‐R) and repeated the experiments. We found that the 95% Lys‐R diet again did not affect food intake (Figure S2a: *p* = 0.38), and it led to a trend toward greater weight loss compared to the 90% Leu‐R diet in B6 mice (Figure S2b: *p* = 0.14). However, the 95% Lys‐R diet failed to reduce small intestinal tumor size (Figure S2c: (< 1 mm) 95% Lys‐R vs. 90% Leu‐R, *p* = 0.0004; (1‐3 mm) 95% Lys‐R vs. 90% Leu‐R, *p* = 0.0002; (> 3 mm) 95% Lys‐R vs. 90% Leu‐R, *p* = 0.001) and number (Figure S2d: *p* < 0.0001) to the extent observed with the 90% Leu‐R diet. Notably, none of the amino acid‐restricted diets significantly influenced colon tumor numbers (Figure 1g, S2e. Figure 1g: F (3,79) =4.58, *p* = 0.0052; AIN vs. 90% Arg‐R, *p* = 0.15; AIN vs. 90% Lys‐R, *p* > 0.99; AIN vs. 90% Leu‐R, *p* = 0.086. Figure S2e: *p* = 0.62). However, the 90% Leu‐R group tended to show a lower number of colon tumors (Figure 1g: AIN vs. 90% Leu‐R, *p* = 0.086) and a higher proportion of tumor‐free mice (Figure 1h: AIN vs. 90% Leu‐R, *p* = 0.078).

A previous study has reported sex‐specific differences in Apc^Min/+^ mice in terms of survival, body mass, and muscle mass (Schrems et al. 2023). To determine whether the tumor‐suppressive effect of 90% Leu‐R in our study was also sex‐specific, we performed separate analyses of tumor numbers in male and female mice in the 90% Leu‐R group. The results showed that tumor numbers were significantly reduced in both male (Figure S3a: *p* < 0.0001) and female (Figure S3b: *p* = 0.0004) mice fed the 90% Leu‐R diet, suggesting that sex was not a major factor influencing the tumor‐suppressive effect of Leu‐R.

These data suggest that different amino acids have distinct effects, with leucine potentially playing a pivotal role in small intestinal tumorigenesis.

### 3.2 Leu‐R Diets (50%, 70%, or 90% in Late‐Stage Adenoma) do Not Affect Intestinal Tumor Size and Number in Apc^Min^ ^/+^ Mice

**FIGURE 2:**
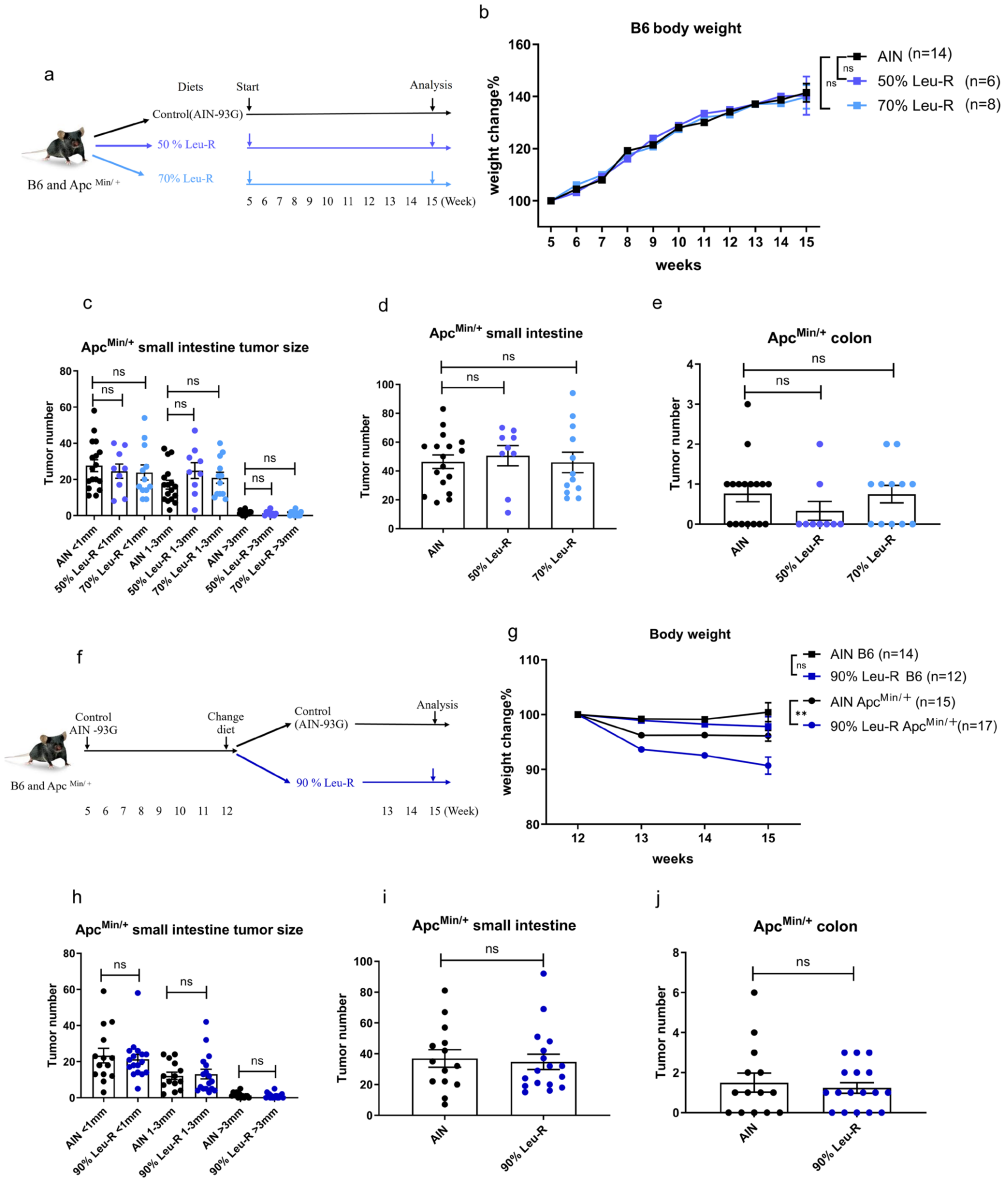
Leu‐R diets (50%, 70%, or 90% in late‐stage adenoma) do not affect intestinal tumor size and number in Apc^Min/+^ mice (a) Feeding schedule of the 50% and 70% Leu‐R diets. (b) Body weight changes were shown, and statistical analysis was performed at week 15. (c) Tumor size and (d) number in the small intestine, and (e) number in the colon. AIN (*n* = 17), 50% Leu‐R (*n* = 8), 70% Leu‐R (*n* = 12). (f) Feeding schedule of the 90% Leu‐R diet in late‐stage adenoma. (g) Body weight changes were shown, and statistical analysis was performed at week 15. (h) Tumor size and (i) number in the small intestine, and (j) number in the colon: AIN (*n* = 14), 90% Leu‐R (*n* = 17). Data are presented as the mean ± SEM; statistical analysis was conducted using an unpaired two‐tailed Student's *t*‐test (Figure 2g–j), or one‐way ANOVA followed by Tukey's multiple‐comparisons test (Figure 2b–e). Statistical significance levels are indicated as follows: Ns (not significant), *p* > 0.05.

To determine whether tumorigenesis correlates with leucine levels, similar experiments were conducted using milder leucine restrictions (50% Leu‐R and 70% Leu‐R) (Figure 2a). The result showed that the 50% and 70% Leu‐R diets did not affect the body weight gain in B6 mice (Figure 2b: F (2,25) = 0.037, *p* = 0.96). The size of small intestinal tumors (Figure 2c: (< 1 mm), F (2,35) = 0.33, *p* = 0.72; (1‐3 mm), F (2,35) = 1.49, *p* = 0.24; (> 3 mm), F (2,35) = 0.26, *p* = 0.78), as well as the number of small intestinal tumors (Figure 2d: F (2,35) = 0.14, *p* = 0.87) and colon tumors (Figure 2e: F (2,35) = 1.02, *p* = 0.37), remained unaffected. This result suggests that there is a threshold of leucine requirements for mouse growth and tumor initiation and/or tumor size growth.

We then wondered whether leucine restriction can slow down tumor growth even after the tumor develops to some extent. To answer this question, we began the Leu‐R diet at 12 weeks of age (Figure 2f). After switching diets, B6 mice in the 90% Leu‐R group exhibited only a slight reduction in body weight (Figure 2g: AIN B6 vs. 90% Leu‐R B6, *p* = 0.054), whereas Apc^Min/+^ mice showed a pronounced tendency toward weight loss (Figure 2g: AIN Apc^Min/+^ vs. 90% Leu‐R Apc^Min/+^, *p* = 0.0035). However, replacing AIN‐93G with the 90% Leu‐R diet during the late tumor stage (~12–15 weeks of age) did not affect small intestinal tumor size (Figure 2h: (< 1 mm) AIN vs. 90% Leu‐R, *p* = 0.67; (1‐3 mm) AIN vs. 90% Leu‐R, p = 0.78; (> 3 mm) AIN vs. 90% Leu‐R, *p* = 0.37) and number (Figure 2i: *p* = 0.77). Colon tumor numbers were unaffected as well (Figure 2j: *p* = 0.62). These data indicate that both the leucine content of the diet and the timing of dietary intervention influence tumorigenesis.

### 3.3 The 90% Leu‐R Diet Does Not Affect Paneth Cell‐Derived Wnt3 Expression or Stem Cells in the Small Intestine

Next, we investigated the mechanism of tumor reduction by the 90% Leu‐R diet. In the small intestinal crypt, Paneth cells secrete Wnt3 to support stem cell growth (Sato, van Es, et al. 2011), and Lgr5^+^ stem cells are considered the origin of Apc‐deficient tumors (Barker et al. 2009). It has been reported that Paneth cell‐specific deletion of Wnt3 leads to a reduction in small intestinal tumors, but not in the colon, of Apc^Min/+^ mice (Chen et al. 2021). Furthermore, we previously showed that deletion of L‐type amino acid transporter 1 (LAT1), which is expressed on the basolateral side of intestinal crypt cells (Kandasamy et al. 2018) and transports leucine but not lysine (Yan et al. 2019), led to the reduction of small intestinal tumorigenesis and reduced tumor cell proliferation (Sui et al. 2023). These effects were associated with reduced Paneth cell numbers and Wnt3 expression, along with suppression of the mTORC1 pathway in Apc^Min/+^ mice (Sui et al. 2023). Since the 90% Leu‐R diet reduced circulating leucine levels (Figure S4: *p* < 0.0001), and leucine is an activator of the mTORC1 pathway (Cangelosi et al. 2022; Wolfson et al. 2016), we hypothesized that the reduction in small intestinal tumors observed in this study may occur through a mechanism similar to that of LAT1‐deficient mice (Sui et al. 2023).

**FIGURE 3:**
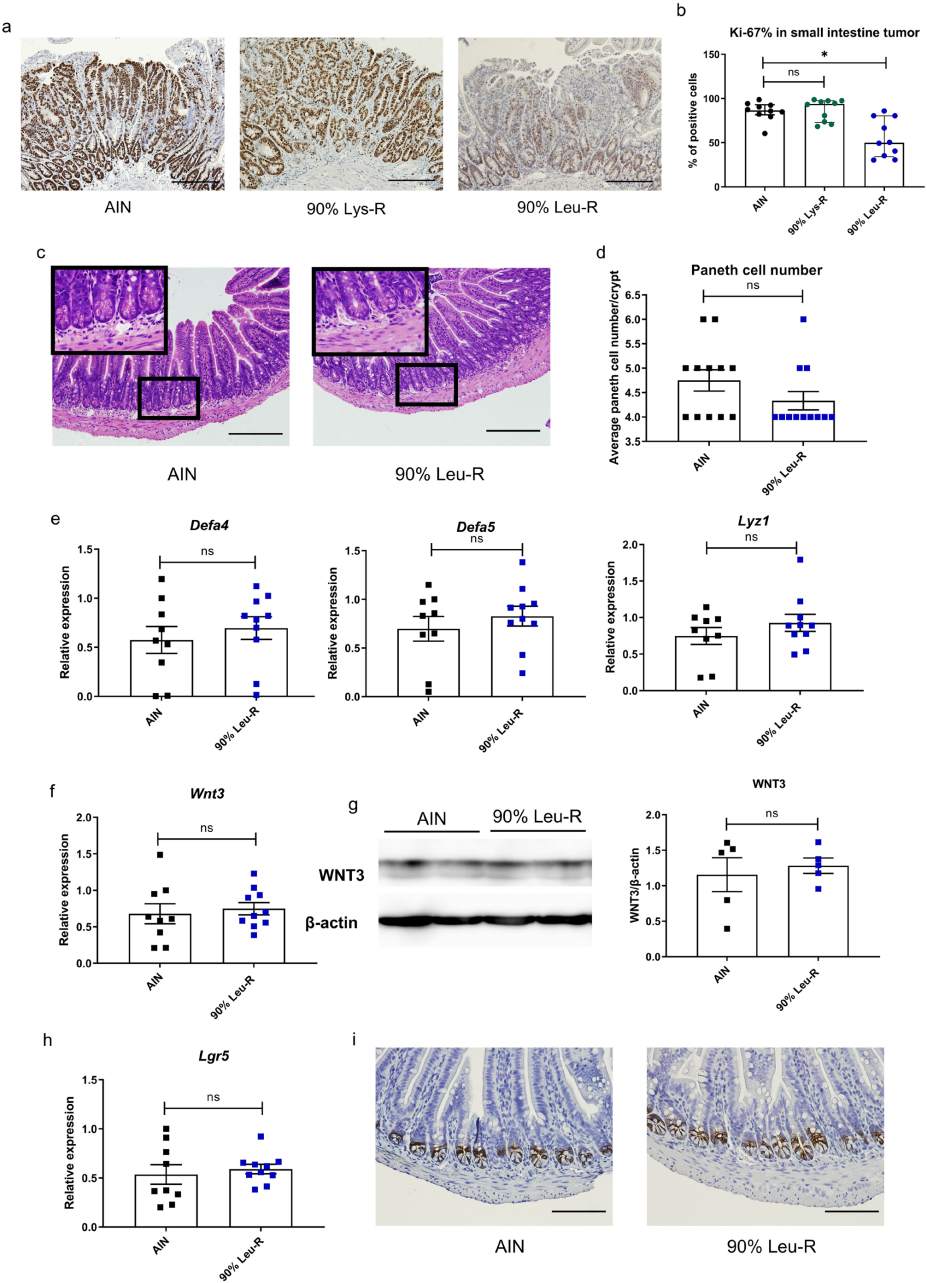
The 90% Leu‐R diet does not affect Paneth cell‐derived Wnt3 expression or stem cells in the small intestine (a) Representative images of Ki‐67 staining in tumors (×200 magnification, scale bar = 200 μm). (b) Ki‐67 positive cells analyzed using ImageJ software: AIN (*n* = 10), 90% Lys‐R (*n* = 10), 90% Leu‐R (*n* = 10). (c) Representative images of HE staining in the small intestine (×200 and ×1000 magnification [inset], scale bar = 200 μm). (d) Average Paneth cell number/crypt: AIN (*n* = 12), 90% Leu‐R (*n* = 12). (e) Gene expression of *Defa4*, *Defa5*, and *Lyz1*: AIN (*n* = 9), 90% Leu‐R (*n* = 10). (f) Gene expression of *Wnt3*: AIN (*n* = 9), 90% Leu‐R (*n* = 10); (g) Protein levels of WNT3: AIN (*n* = 5), 90% Leu‐R (*n* = 5). (h) Gene expression of *Lgr5*: AIN (*n* = 9), 90% Leu‐R (*n* = 10). (i) Immunohistochemical staining of Olfm4 (200× magnification), scale bar = 200 μm. Data in Figure 3b are presented as median with IQR, whereas other data (Figure 3d–h) are presented as mean ± SEM; statistical analysis was performed using an unpaired two‐tailed Student's *t*‐test (Figure 3d–h), and Kruskal–Wallis test followed by Dunn's multiple‐comparisons test (Figure 3b). Statistical significance levels are indicated as follows: ***p < 0.001, ns (not significant), *p* > 0.05.

Thus, we first performed Ki‐67 staining to assess cell proliferation and evaluated mTORC1 activity by staining for phosphorylated S6 (p–S6), a downstream target of mTORC1. The results revealed a decreased number of Ki‐67‐positive cells (Figure 3a,b: AIN vs. 90% Leu‐R, *p* = 0.013) and reduced mTORC1 pathway activation in tumors from mice fed the 90% Leu‐R diet (Figure S5a,b: AIN vs. 90% Leu‐R, *p* = 0.022). In contrast, the 90% Lys‐R diet had no significant effect on tumor cell proliferation (Figure 3a,b: AIN vs. 90% Lys‐R, *p* > 0.99). We then counted the number of Paneth cells using HE‐stained sections and examined the expression of Paneth cell markers (*Defa4*, *Defa5*, and *Lyz1*) by real‐time PCR. However, the 90% Leu‐R diet did not affect Paneth cell numbers (Figure 3c,d: AIN vs. 90% Leu‐R, *p* = 0.16) or the expression levels of Paneth cell markers (Figure 3e: *Defa4*, *p* = 0.51; *Defa5*, *p* = 0.43; *Lyz1*, *p* = 0.29). We further confirmed that Wnt3 expression was unaffected at both mRNA (Figure 3f: *p* = 0.67) and protein levels (Figure 3h: *p* = 0.64) in the 90% Leu‐R group. Additionally, intestinal stem cells, assessed by *Lgr5* gene expression (Figure 3h: *p* = 0.61) and immunohistochemical staining for Olfm4 (van der Flier et al. 2009) (Figure 3i), showed no significant changes. These findings indicate that a 90% Leu‐R diet inhibits tumor cell proliferation and affects tumor growth in the small intestine of Apc^Min/+^ mice, likely through inhibition of the mTORC1 pathway. However, it does not affect small intestinal tumorigenesis through Paneth cell‐derived Wnt3 expression or reduced number of intestinal stem cells.

### 3.4 The 90% Leu‐R Diet Affects Biological Processes in Tumors, Including DNA Repair and Genome Stability

**FIGURE 4:**
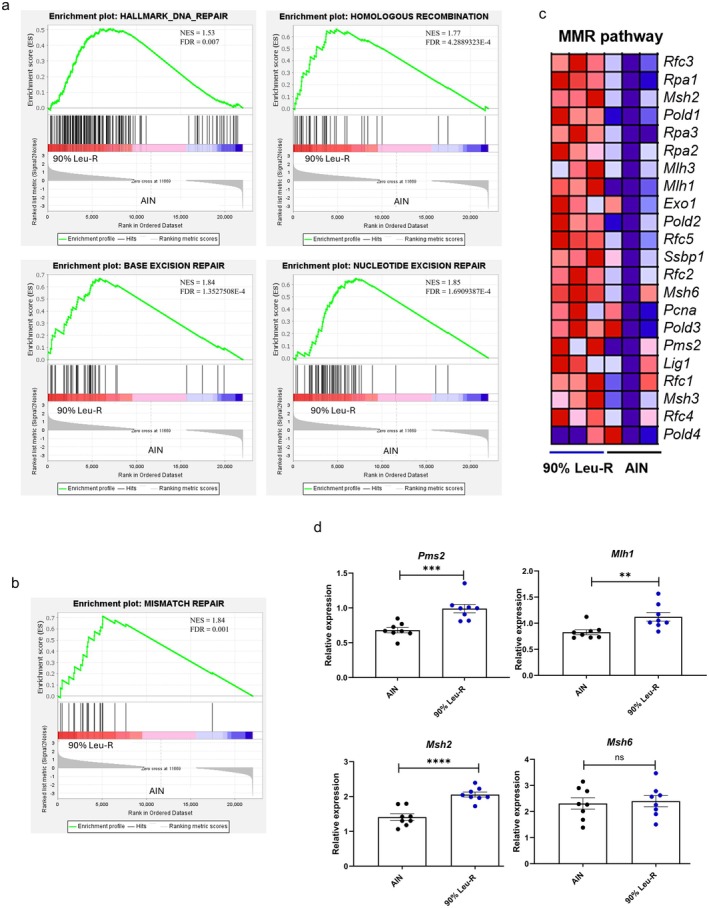
The 90% Leu‐R diet affects biological processes in tumors, including DNA repair and genome stability (a) GSEA for genes related to DNA repair and genome stability in tumors. (b) GSEA for genes related to the MMR pathway, and (c) heatmap of the MMR pathway genes in tumors, with red indicating upregulation and blue indicating downregulation. AIN (*n* = 3), 90% Leu‐R (*n* = 3). (d) Expression of several core genes of the MMR pathway in tumors: AIN (*n* = 8), 90% Leu‐R (*n* = 8). Data are presented as the mean ± SEM; statistical analysis was performed using an unpaired two‐tailed Student's *t*‐test. Statistical significance levels are indicated as follows: **p < 0.01, ***p < 0.001, ****p < 0.0001; ns (not significant), *p* > 0.05.

Subsequently, GSEA was performed to investigate the effects of the 90% Leu‐R diet on the molecular characteristics and biological behavior of tumors. Among the results (Table S2), gene sets associated with DNA‐related biological processes, including DNA repair and genome stability maintenance, were significantly enriched in tumors from the 90% Leu‐R group (Figure 4a). Moreover, the mismatch repair (MMR) pathway was also significantly enriched in this group (Figure 4b), and the heatmap showed increased expression levels of several genes associated with the MMR (Figure 4c). MMR is a crucial DNA repair system responsible for correcting base mismatches during DNA replication, thereby ensuring genomic stability and preventing the accumulation of mutations (He et al. 2022; Pećina‐Šlaus et al. 2020). Loss of MMR is shown to exacerbate intestinal tumorigenesis in Apc^Min/+^ mice (Haigis et al. 2004; Reitmair et al. 1996; Shoemaker et al. 2000). To confirm this finding, we measured the expression levels of several core MMR genes, such as *Pms2*, *Mlh1*, *Msh2*, and *Msh6*, by real‐time PCR. The results showed that the expression levels of *Pms2*, *Mlh1*, and *Msh2* remained unchanged in small intestinal normal tissues (Figure S6: *Pms2*, *p* = 0.15; *Mlh1*, *p* = 0.36; *Msh2*, *p* = 0.43; *Msh6*, *p* = 0.44) but were significantly higher in the tumors from the 90% Leu‐R group (Figure 4d: *Pms2*, *p* = 0.0007; *Mlh1*, *p* = 0.008; *Msh2*, *p* < 0.0001; *Msh6*, *p* = 0.77), whereas no difference was observed in the tumors from the 90% Lys‐R group (Figure S7: *Pms2*, *p* = 0.61; *Mlh1*, *p* = 0.78; *Msh2*, *p* = 0.51; *Msh6*, *p* = 0.90). This suggests that Leu‐R may inhibit the initiation of tumor development and/or excessive cell proliferation by enhancing MMR‐mediated DNA repair and preventing mutation accumulation.

### 3.5 Organoids Derived From Small Intestinal Tumors in Mice Fed the 90% Leu‐R Diet Exhibit Fewer and Smaller Spherical Structures

We utilized intestinal organoids for a detailed investigation. Tumor‐derived organoids, such as those from Apc‐deficient adenomas, generally form simple spherical cysts (Sato et al. 2009; Sato, Stange, et al. 2011). In contrast, normal intestinal organoids exhibit crypt‐like budding structures. In Apc^Min/+^ mice, tumor formation occurs when the remaining wild‐type *Apc* allele acquires additional mutations, resulting in loss of heterozygosity (LOH) (Luongo et al. 1994). Therefore, we hypothesized that if the frequency of LOH is reduced, the organoids from the 90% Leu‐R group should show a reduced number of spherical organoids.

**FIGURE 5:**
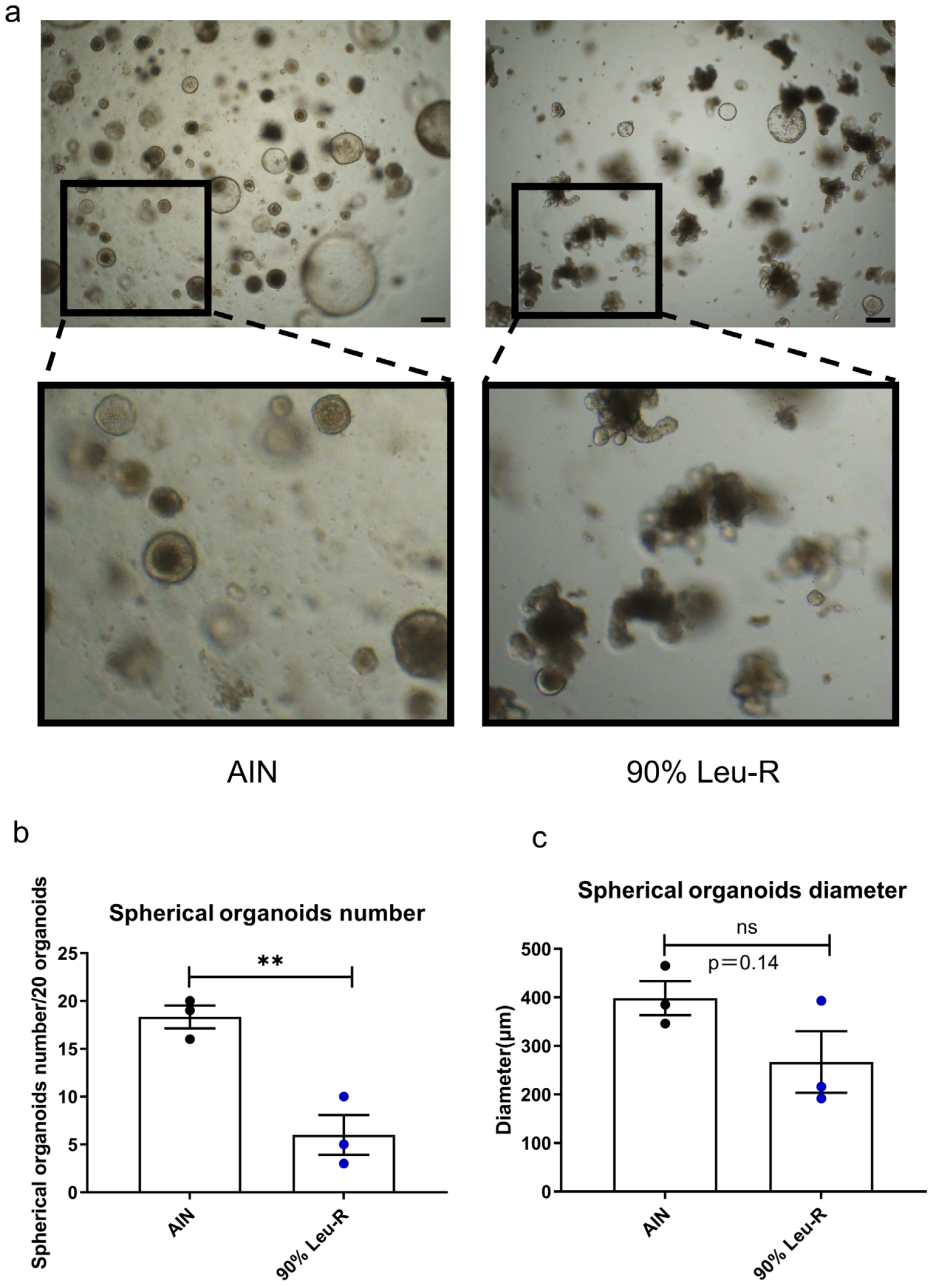
Organoids derived from small intestinal tumors in mice fed the 90% Leu‐R diet exhibit fewer and smaller spherical structures (a) Representative images on day 5 (×40 magnification, scale bar = 200 μm). The boxed region is digitally enlarged to facilitate visualization of organoid morphology. (b) The number and (c) size of Apc^Min/+^ (spherical) organoids on day 5: AIN (*n* = 3), 90% Leu‐R (*n* = 3). Data are presented as the mean ± SEM; statistical analysis was conducted using an unpaired two‐tailed Student's *t*‐test. Statistical significance levels are indicated as follows: **p < 0.01, ns (not significant), *p* > 0.05.

As expected, representative images suggested that organoids from the 90% Leu‐R group appeared more likely to form crypt‐like budding structures (Figure 5a). More importantly, these organoids also formed significantly fewer spherical structures (Figure 5a,b; *p* = 0.0068), with a trend toward smaller size (Figure 5a,c; *p* = 0.14). Combined with the GSEA results showing enrichment of gene sets related to DNA repair and genome stability maintenance in tumors from the 90% Leu‐R group, our findings suggest that the 90% Leu‐R diet may be associated with reduced LOH and preservation of Apc gene stability to suppress small intestinal tumorigenesis.

Taken together, our data demonstrate that leucine plays a key role in small intestinal tumorigenesis in Apc^Min/+^ mice. A 90% Leu‐R diet reduced the activation of the mTORC1 pathway. It also increased the expression of MMR genes, which may maintain genomic stability, and conferred a reduction in cell proliferation, thereby attenuating small intestinal tumorigenesis.

## 4 Discussion

Leucine has been reported to possess both anti‐ and pro‐tumorigenic properties depending on dose, cancer type, metabolic context, intervention timing, and other factors (Akbay et al. 2024). In the present study, we found that a Leu‐R diet significantly reduced both the size and number of small intestinal tumors in Apc^Min/+^ mice, whereas restriction of other amino acids, such as lysine and arginine, did not confer similar tumor‐suppressive effects. Notably, the antitumor effect of the Leu‐R diet was evident only under more stringent restriction (90%) and when implemented at early stages of tumor development.

Leucine serves as a key regulator of the mTORC1 pathway via Sestrin2‐GATOR2‐Rag‐GTPase signaling (Cangelosi et al. 2022; Wolfson et al. 2016). Under leucine‐rich conditions, Sestrin2 is released from GATOR2, leading to RAG‐GTPase and subsequent mTORC1 activation, which promotes cell proliferation (Cangelosi et al. 2022; Wolfson et al. 2016). Another essential amino acid, lysine, does not directly bind Sestrin2 to modulate mTORC1 activity (Dyachok et al. 2016; Valenstein et al. 2022). This difference may underscore the leucine‐specific effects observed in our study, emphasizing that not all essential amino acids exert an equal influence on intestinal tumorigenesis. Besides lysine, we tested whether the observed leucine‐specific effects could be generalized to other mTORC1‐regulating amino acids, such as valine and arginine (Dyachok et al. 2016). However, the 90% Val‐R diet markedly reduced appetite and body weight, which is consistent with a previous report (Jin et al. 2025), and the 90% Arg‐R diet did not affect the tumor size or number in our setting, possibly because arginine is a non‐essential amino acid. It would be interesting to investigate whether a mild reduction in valine concentration, or targeted deletion of arginine transporters (e.g., CAT‐1; Du and Han 2021) in the intestinal epithelium, could modulate intestinal tumorigenesis without affecting appetite or completely blocking arginine uptake into tumors, respectively.

Paneth cells and Wnt3 expression modulate small intestinal tumorigenesis in Apc^Min/+^ mice (Chen et al. 2021; Sui et al. 2023). LAT1 mediates leucine transport (Yan et al. 2019), and its deletion in the intestinal epithelium reduces Paneth cell numbers and Wnt3 expression, thereby affecting small intestinal tumorigenesis (Sui et al. 2023). We initially hypothesized that the tumor‐suppressive effect of the Leu‐R diet is mediated by a reduction in Paneth cell number and Wnt3 expression, similar to the effects observed following LAT1 deletion. However, such reductions were not observed in mice fed the Leu‐R diet. This suggests that a 10% leucine content in the food may be enough to develop Paneth cells. It is also possible that neutral amino acids other than leucine, transported by LAT1, may play a crucial role in Paneth cell formation. In any case, the reduction in small intestinal tumor formation observed in this study appears to involve a mechanism distinct from that seen in the LAT1 conditional knockout mice (Sui et al. 2023).

In Apc^Min/+^ mice, mutation of the remaining wild‐type Apc allele leads to LOH, thereby promoting adenoma development (Luongo et al. 1994). The MMR pathway plays a critical role in preventing the accumulation of mutations (He et al. 2022; Pećina‐Šlaus et al. 2020), and its deficiency has been shown to exacerbate intestinal tumorigenesis in Apc^Min/+^ mice (Haigis et al. 2004; Reitmair et al. 1996; Shoemaker et al. 2000). Our results showed that in the small intestinal tumors from the 90% Leu‐R group, the expression levels of *Mlh1*, *Msh2*, and *Pms2* were significantly higher, and the enriched gene sets were primarily associated with DNA‐related biological processes, including DNA repair and maintenance of genomic stability. This suggests that under Leu‐R conditions, tumors maintain DNA repair mechanisms via the MMR system in response to reduced proliferation. This might maintain genomic stability, reduce Apc LOH, and ultimately attenuate intestinal tumorigenesis. Supporting this, organoids derived from small intestinal tumors in mice fed a 90% Leu‐R diet not only exhibited fewer and smaller spherical structures but also developed morphologies resembling normal crypt‐like budding.

One surprising result is that, although a 90% Leu‐R diet significantly slowed down tumor cell proliferation as shown by Ki‐67 staining, switching from control to 90% Leu‐R chow at 12 weeks of age did not suppress tumor growth in Apc^Min/+^ mice at all (Figure 2f,h–j). Notably, this late‐stage dietary intervention led to more pronounced body weight loss in Apc^Min/+^ mice than in B6 mice, which were subjected to the same dietary switch (Figure 2g). This finding may suggest that, once a tumor develops, nutrient deprivation (e.g., Leu‐R) may trigger catabolism of host tissues (e.g., skeletal muscle) to supply essential nutrients, which may in turn be preferentially directed to or utilized by the tumors. This phenomenon closely resembles cancer cachexia, a condition commonly observed in terminal‐stage cancer patients, characterized by continued tumor growth accompanied by severe body weight and muscle mass loss (Berriel Diaz et al. 2024; Fearon et al. 2011). Given that muscle wasting is a key feature of cachexia, it is intriguing to consider that the reduction in serum leucine levels caused by the 90% Leu‐R diet might trigger leucine mobilization from muscle tissue. However, this possibility warrants further investigation.

Moreover, we found that the 90% Leu‐R diet had no significant effect on colon tumors, despite reducing blood leucine levels. This suggests distinct mechanisms between colonic and small intestinal tumorigenesis, and that blood leucine levels alone are insufficient to account for their phenotypic differences. The small intestine is the primary organ for digestion and absorption, and the amino acid content of the small intestine is much higher than that of the colon (Bröer 2023). Amino acids in the contents of the small intestine may directly contribute to tumorigenesis, making small intestinal tumors more sensitive to Leu‐R. Additionally, the proliferation rate of the small intestinal epithelial cells is much higher than that of the colonic epithelial cells (Assumpção et al. 2020). It is also possible that Leu‐R reduces tumorigenesis by suppressing the proliferation rate of small intestinal epithelial cells, thereby allowing sufficient time for DNA repair. Overall, further in‐depth studies are required to investigate the mechanisms by which Leu influences colon tumorigenesis.

Despite these insights, our study has limitations. Although leucine restriction was associated with both reduced mTORC1 activity and increased expression of MMR‐related genes, the present study does not establish a causal relationship between mTORC1 suppression and MMR upregulation. Therefore, we cannot conclude whether the induction of MMR genes is directly mediated by reduced mTORC1 signaling or occurs through an mTORC1‐independent mechanism. Further studies are required to determine whether mTORC1 suppression directly contributes to the upregulation of MMR genes under leucine‐restricted conditions. We did not directly assess Apc LOH status in tumors from mice in the Leu‐R group. Organoids derived from tumors of mice fed the 90% Leu‐R diet showed a trend of fewer spherical structures and smaller diameter. However, the small sample size reduces the statistical power of this analysis. Further studies using larger independent organoid cohorts are warranted to validate these findings. APC mutation‐driven malignancies, such as those occurring in patients with familial adenomatous polyposis (FAP), are primarily observed in the colon in clinical settings. Thus, the applicability of the present findings to human colorectal cancer remains unclear and requires further investigation using relevant colon cancer models.

## 5 Conclusion

In summary, different amino acids exert distinct effects on small intestinal tumorigenesis, with leucine potentially playing a pivotal role in Apc^Min/+^ mice. The 90% Leu‐R diet reduced the tumor size and number in the small intestine of Apc^Min/+^ mice by reducing tumor cell proliferation at least partly via reduced mTORC1 pathway activation. Tumor‐derived organoids from the 90% Leu‐R diet group showed restored formation of crypt‐like budding structures, supporting the idea of the promotion of DNA repair mechanisms through the MMR system in this group. However, as this mechanism has not been directly demonstrated, it should be regarded as a hypothesis‐generating observation. Further investigation, including direct analysis of Apc LOH in response to the 90% Leu‐R diet, is warranted. These findings provide a theoretical basis for the development of dietary leucine modulation strategies to prevent intestinal tumorigenesis.

## Author Contributions

**Makoto Ooi:** formal analysis. **Yunlong Sui:** methodology, formal analysis, writing – original draft, writing – review and editing, investigation. **Daisuke Watanabe:** formal analysis, investigation. **Hui Yang:** writing – review and editing, resources. **Yuna Ku:** formal analysis, investigation. **Misaki Agawa:** formal analysis, investigation. **Haruka Miyazaki:** formal analysis, investigation. **Hirotaka Nakamura:** formal analysis, investigation. **Norihiro Okamoto:** formal analysis, investigation. **Jun Inoue:** formal analysis, investigation. **Yuzo Kodama:** writing – review and editing, resources, supervision. **Namiko Hoshi:** methodology, formal analysis, writing – original draft, writing – review and editing, funding acquisition, investigation, resources.

## Funding

Japan Society for the Promotion of Science (23K18005).

## Ethics Statement

This study was approved by the Institutional Animal Care and Use Committee of Kobe University (approval number: P190306).

## Conflicts of Interest

The authors declare no conflicts of interest.

## Supporting information


**Figure S1:** The 90% Val‐R diet significantly reduces appetite and body weight.
**Figure S2:** The 95% Lys‐R diet does not reduce the small intestinal tumor size and number to the level observed with the 90% Leu‐R diet.
**Figure S3:** The 90% Leu‐R diet reduces tumor number in both male and female ApcMin/+ mice.
**Figure S4:** The 90% Leu‐R diet limits leucine availability in the blood.
**Figure S5:** The 90% Leu‐R diet suppresses the mTORC1 pathway activation in tumors.
**Figure S6:** The 90% Leu‐R diet does not affect the gene expression of MMR in small intestinal normal tissue. MMR gene expression in small intestines.
**Figure S7:** The 90% Lys‐R diet does not affect the gene expression of MMR in ApcMin/+ tumors.

**Table S1:** The nutritional composition of different types of diets.

**Table S2:** Gene sets enriched in phenotype 90% Leu‐R tumors.

## Data Availability

The data that support the findings of this study are available from the corresponding author upon reasonable request.
