# Supplementary material for: Leucine‐Restricted Diet Attenuates Small Intestinal Tumorigenesis in ApcMin /+ Mice
Source: Food Sci Nutr. 2026 Jul 9;14(7):e72100. doi: 10.1002/fsn3.72100 (PMC13349113; doi:10.1002/fsn3.72100)
Supplement: Supplementary file 1 — Figure S1: The 90% Val‐R diet significantly reduces appetite and body weight. Figure S2: The 95% Lys‐R diet does not reduce the small intestinal tumor size and number to the level observed with the 90% Leu‐R diet. Figure S3: The 90% Leu‐R diet reduces tumor number in both male and female ApcMin/+ mice. Figure S4: The 90% Leu‐R diet limits leucine availability in the blood. Figure S5: The 90% Leu‐R diet suppresses the mTORC1 pathway activation in tumors. Figure S6: The 90% Leu‐R diet does not affect the gene expression of MMR in small intestinal normal tissue. MMR gene expression in small intestines. Figure S7: The 90% Lys‐R diet does not affect the gene expression of MMR in ApcMin/+ tumors. [file FSN3-14-e72100-s003.docx]

**Supplementary figures and legends**


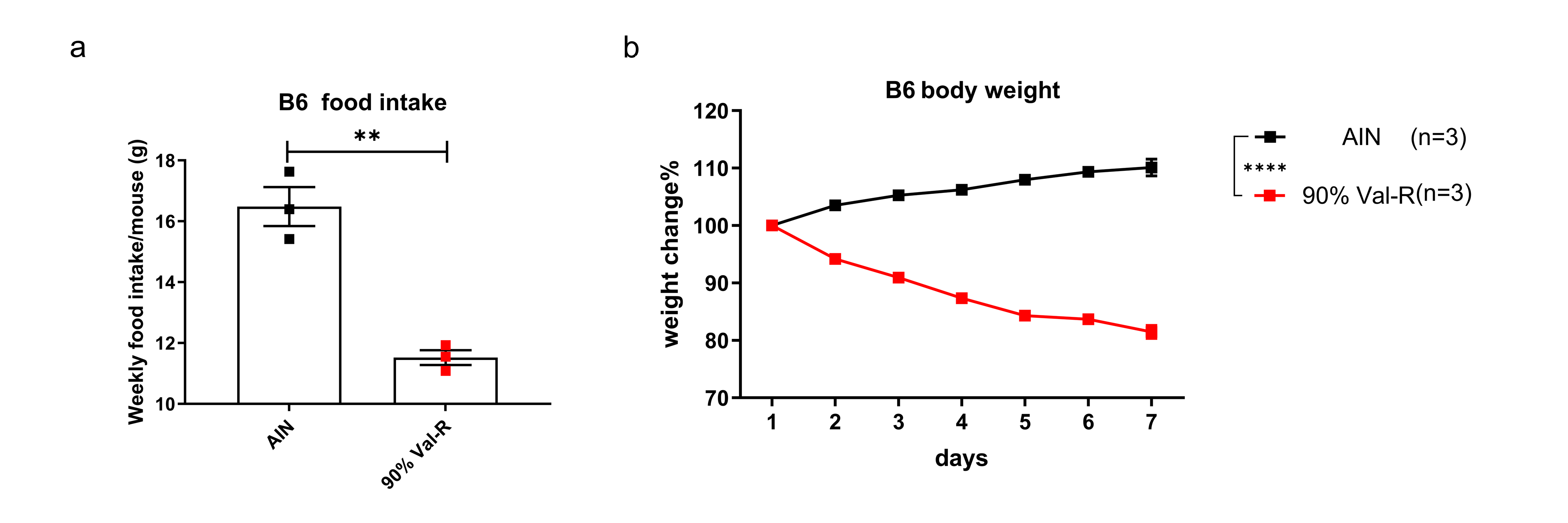


**Fig. S1** The 90% Val-R diet significantly reduces appetite and body weight. Body weight was monitored daily for one week, and food intake was measured over the same one-week period. (a) The weekly food intake. Mice were housed individually in single cages. (b) Body weight changes were shown, and statistical analysis was performed at day 7. Data are presented as the mean ± SEM; statistical analysis was conducted using an unpaired two-tailed Student’s t-test. Statistical significance levels are indicated as follows: **p < 0.01, ****p < 0.0001


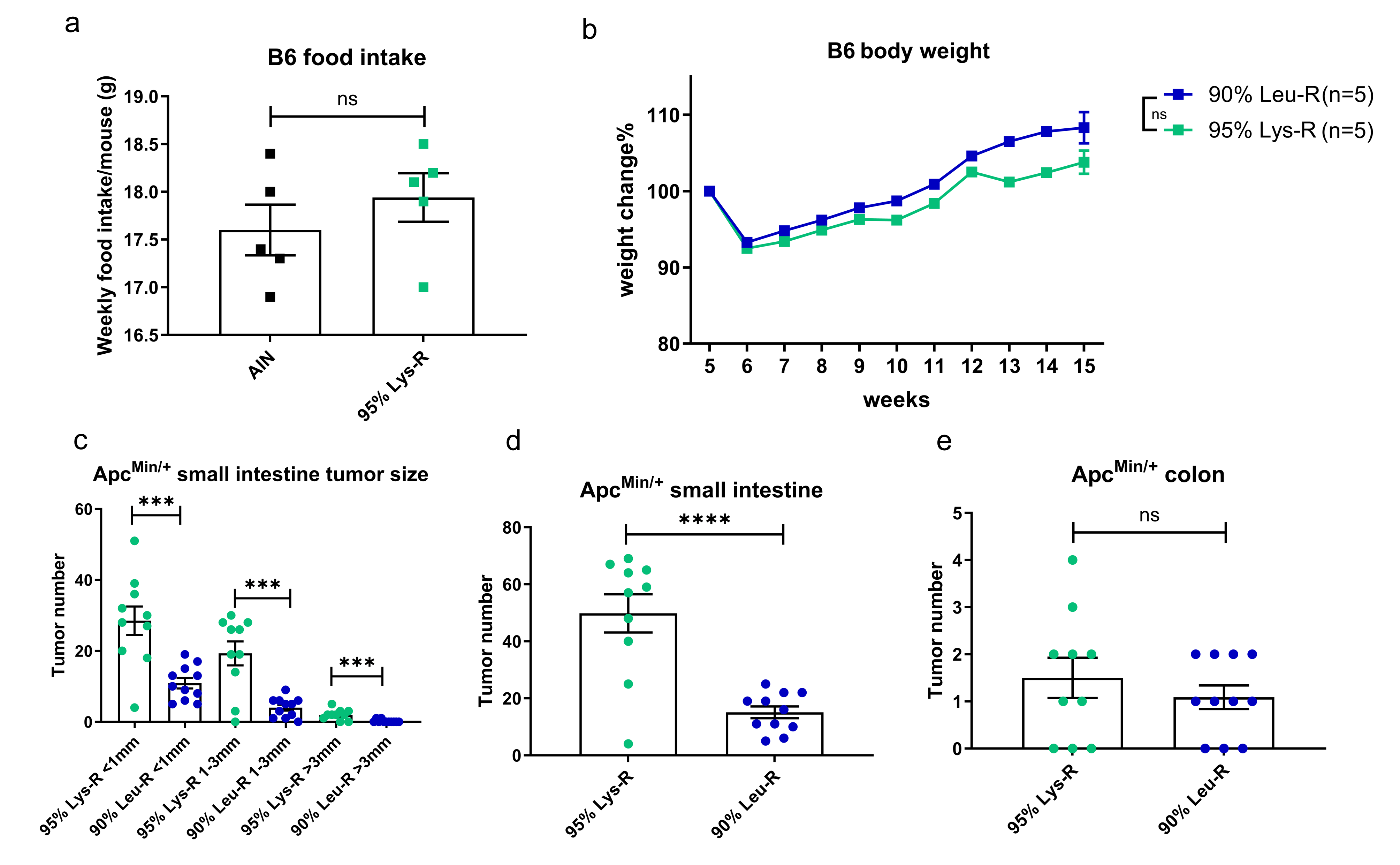


**Fig. S2** The 95% Lys-R diet does not reduce the small intestinal tumor size and number to the level observed with the 90% Leu-R diet. (a) The weekly food intake. Mice were housed individually in single cages. (b) Body weight changes were shown, and statistical analysis was performed at week 15. (c) Tumor size and (d) number in the small intestine, and (e) number in the colon. 95% Lys-R (n=10), 90% Leu-R (n=11). Data are presented as the mean ± SEM; Statistical analyses were performed using an unpaired two-tailed Student's t-test. Statistical significance levels are indicated as follows: ***p < 0.001, ****p < 0.0001, ns (not significant), p > 0.05


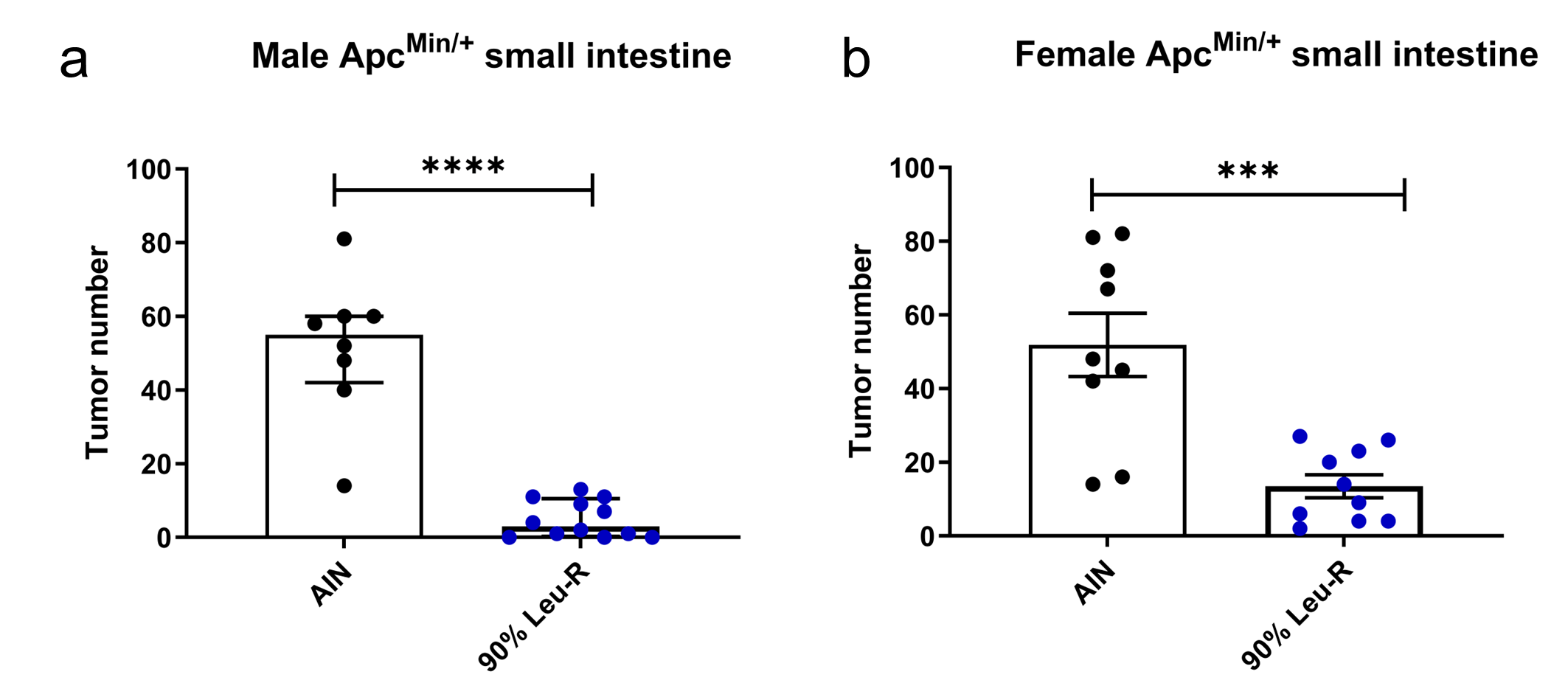


**Fig. S3** The 90% Leu-R diet reduces tumor number in both male and female Apc^Min/+^ mice. (a) Tumor number in male Apc^Min/+^ mice. AIN (n=8), 90% Leu-R (n=12). (b) Tumor number in female Apc^Min/+^ mice. AIN (n=9), 90% Leu-R (n=10). Data are presented as median with IQR (Fig. S3a) and the mean ± SEM (Fig. S3b); Statistical analyses were performed using the Mann–Whitney U test (Fig. S3a) and an unpaired two-tailed Student's t-test (Fig. S3b). Statistical significance levels are indicated as follows: ***p < 0.001, ****p < 0.0001


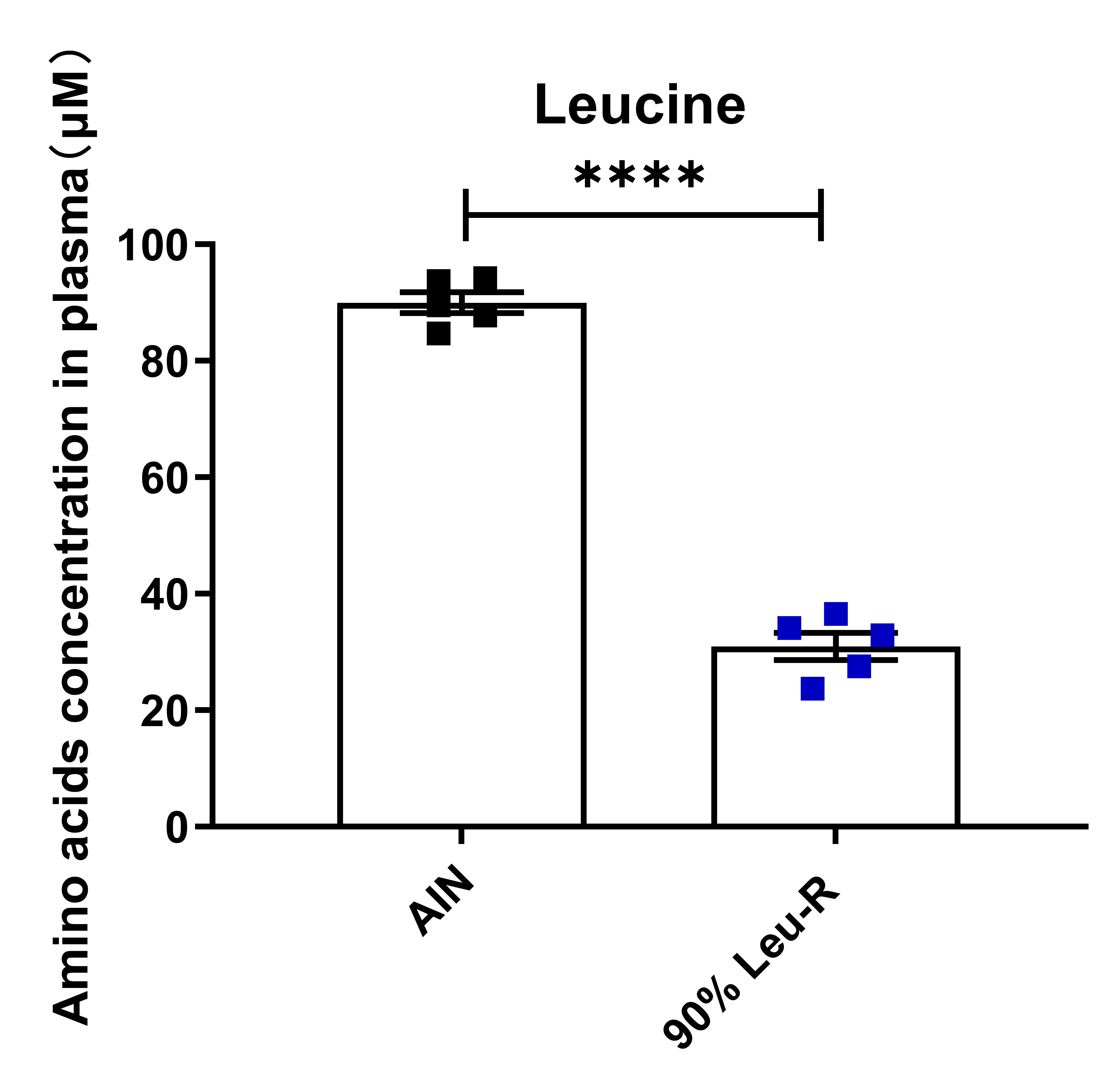


**Fig. S4** The 90% Leu-R diet limits leucine availability in the blood. Plasma leucine concentration analyzed in each group: AIN (n=5), 90% Leu-R (n=5). Data are presented as the mean ± SEM; Statistical analyses were performed using an unpaired two-tailed Student's t-test. Statistical significance levels are indicated as ****p < 0.0001


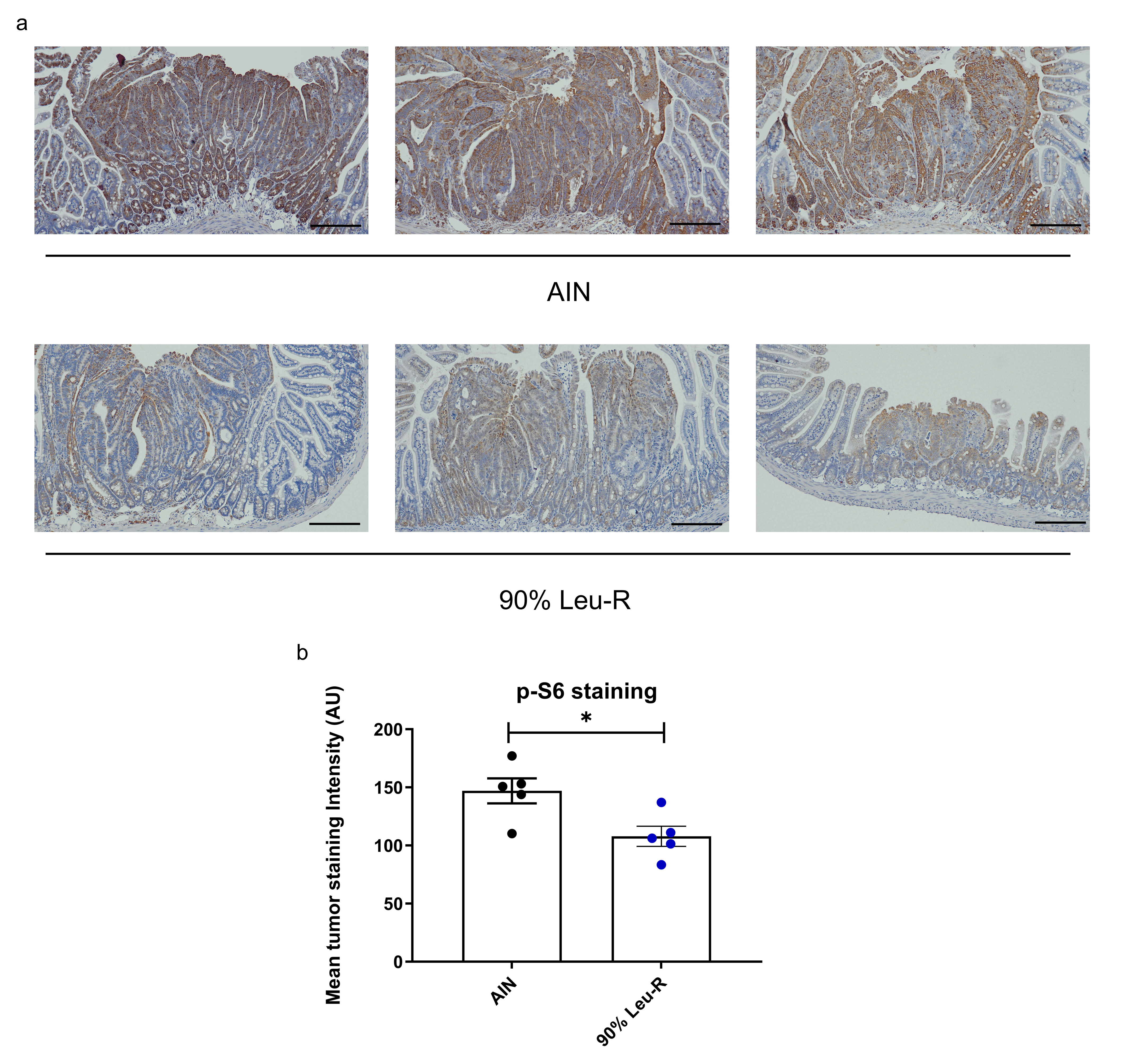


**Fig. S5** The 90% Leu-R diet suppresses the mTORC1 pathway activation in tumors. (a) Staining for p-S6 was performed, and some representative images (×200 magnification) were shown from each group, scale bar=200 μm. (b) p-S6 immunostaining intensity was quantified. Data are presented as the mean ± SEM; Statistical analyses were performed using an unpaired two-tailed Student's t-test. Statistical significance levels are indicated as *p < 0.05


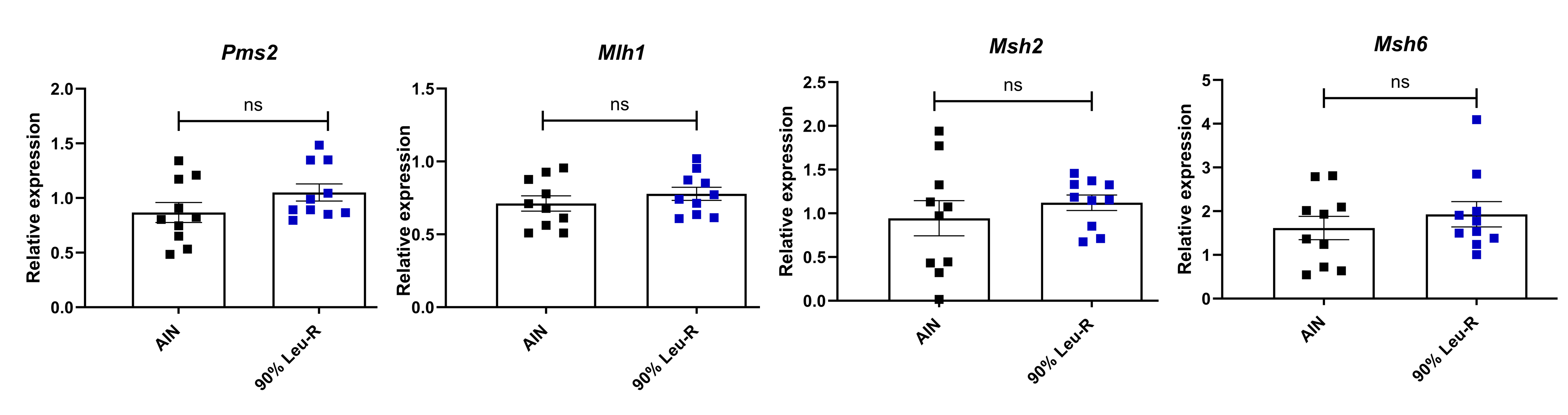
**Fig. S6** The 90% Leu-R diet does not affect the gene expression of MMR in small intestinal normal tissue. MMR gene expression in small intestines. AIN (n=10), 90% Leu-R (n=10). Data are presented as the mean ± SEM; statistical analysis was performed using an unpaired two-tailed Student’s t-test. Statistical significance levels are indicated as follows: ns (not significant); p > 0.05

**
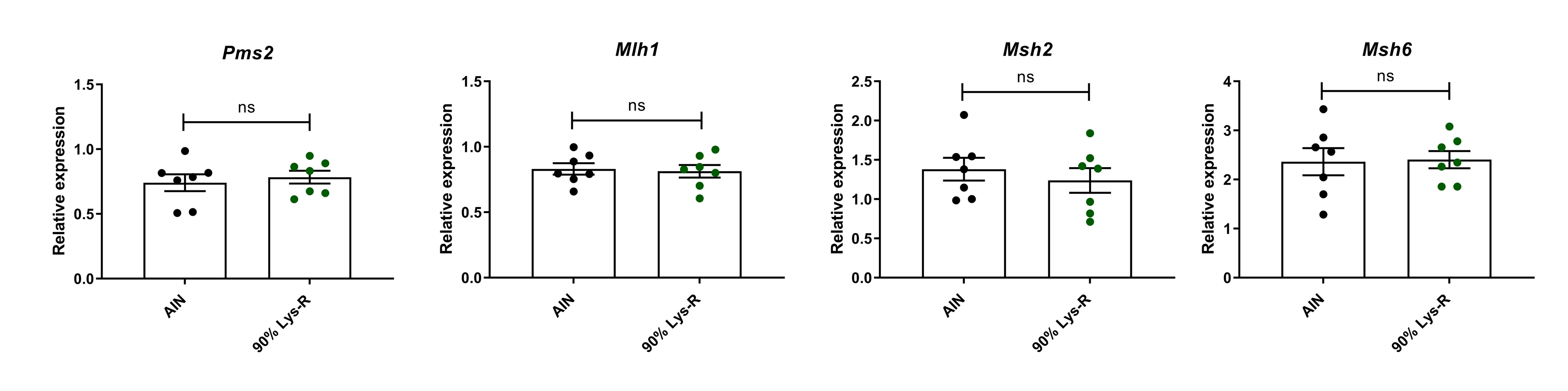
**

**Fig. S7** The 90% Lys-R diet does not affect the gene expression of MMR in Apc^Min/+^ tumors. MMR gene expression in small intestinal tumors. AIN (n=7), 90% Lys-R (n=7). Data are presented as the mean ± SEM; statistical analysis was performed using an unpaired two-tailed Student’s t-test. Statistical significance levels are indicated as follows: ns (not significant); p > 0.05
