# Supplementary material for: Leucine‐Restricted Diet Attenuates Small Intestinal Tumorigenesis in ApcMin /+ Mice
Source: Food Sci Nutr. 2026 Jul 9;14(7):e72100. doi: 10.1002/fsn3.72100 (PMC13349113; doi:10.1002/fsn3.72100)
Supplement: Supplementary file 2 — Table S1: The nutritional composition of different types of diets. [file FSN3-14-e72100-s001.docx]

**Supplementary Table 1** The nutritional composition of different types of diets

| Composition | Unit | AIN-93G | 90% Leu-R | 90% Lys-R | 90% Arg-R | 90% Val-R | 95% Lys-R | 50% Leu-R | 70% Leu-R |
| --- | --- | --- | --- | --- | --- | --- | --- | --- | --- |
| Moisture  Crude Protein  Crude Fat  Crude Ash  Crude Fiber  Nitrogen-Free Extract  Total Calories  Ca  P  Mg  Na  K  Isoleucine  Leucine  Lysine  Methionine  Cystine  Phenylalanine  Tyrosine  Threonine  Tryptophan  Valine  Histidine  Arginine  Alanine  Aspartic Acid  Glutamic Acid  Glycine  Proline  Serine | %  %  %  %  %  %  kcal  %  %  %  %  %  %  %  %  %  %  %  %  %  %  %  %  %  %  %  %  %  %  % | 9.0  18.1  7.3  3.1  5.0  57.6  368.0  0.52  0.32  0.05  0.10  0.36  0.9210  1.5950  1.7050  0.5100  0.3820  0.8710  0.9530  0.7070  0.2140  1.1510  0.5100  0.6250  0.5100  1.2000  3.6160  0.3120  1.9730  0.8880 | 9.0  16.9  7.1  2.7  4.9  59.4  369.2  0.49  0.16  0.05  0.10  0.35  0.9210  0.1595  1.7050  0.5100  0.3820  0.8710  0.9530  0.7070  0.2140  1.1510  0.5100  0.6250  0.5100  1.2000  3.6160  0.3120  1.9730  0.8880 | 9.0  16.8  7.1  2.7  4.9  59.5  369.2  0.49  0.16  0.05  0.10  0.35  0.9210  1.5950  0.1705  0.5100  0.3820  0.8710  0.9530  0.7070  0.2140  1.1510  0.5100  0.6250  0.5100  1.2000  3.6160  0.3120  1.9730  0.8880 | 9.0  17.7  7.1  2.7  4.9  58.6  369.2  0.49  0.16  0.05  0.10  0.35  0.9210  1.5950  1.7050  0.5100  0.3820  0.8710  0.9530  0.7070  0.2140  1.1510  0.5100  0.0625  0.5100  1.2000  3.6160  0.3120  1.9730  0.8880 | 9.0  17.3  7.1  2.7  4.9  59.0  369.2  0.49  0.16  0.05  0.10  0.35  0.9210  1.5950  1.7050  0.5100  0.3820  0.8710  0.9530  0.7070  0.2140  0.1151  0.5100  0.6250  0.5100  1.2000  3.6160  0.3120  1.9730  0.8880 | 9.0  16.7  7.1  2.7  4.9  59.5  369.2  0.49  0.16  0.05  0.10  0.35  0.9210  1.5950  0.0853  0.5100  0.3820  0.8710  0.9530  0.7070  0.2140  1.1510  0.5100  0.6250  0.5100  1.2000  3.6160  0.3120  1.9730  0.8880 | 9.0  17.5  7.1  2.7  4.9  58.8  369.2  0.49  0.16  0.05  0.10  0.35  0.9210  0.7975  1.7050  0.5100  0.3820  0.8710  0.9530  0.7070  0.2140  1.1510  0.5100  0.6250  0.5100  1.2000  3.6160  0.3120  1.9730  0.8880 | 9.0  17.2  7.1  2.7  4.9  59.1  369.2  0.49  0.16  0.05  0.10  0.35  0.9210  0.4785  1.7050  0.5100  0.3820  0.8710  0.9530  0.7070  0.2140  1.1510  0.5100  0.6250  0.5100  1.2000  3.6160  0.3120  1.9730  0.8880 |
